# Thermally Healable Polyurethane Elastomers Based on Biomass Polyester Polyol from Isosorbide and Dimer Fatty Acid

**DOI:** 10.3390/polym16243571

**Published:** 2024-12-20

**Authors:** Se-Ra Shin, Dai-Soo Lee

**Affiliations:** 1 Research Institute, Jungwoo Fine Co., Ltd., #63-8, Seogam-ro 1-gil, Iksan-si 54586, Republic of Korea; 2 C & S Partner, Hanam Technovalley U1 Center, Hanam-si 12982, Republic of Korea; dslee@jbnu.ac.kr

**Keywords:** isosorbide, dimer fatty acid, bio-based polyester polyol, polyurethane elastomer, self-healing

## Abstract

A fully bio-based polyester polyol based on isosorbide (ISB) and dimer fatty acid (DA) was synthesized through esterification. An ISB-based polyester polyol (DIS) was developed to synthesize a bio-based polyurethane elastomer (PUE) with enhanced mechanical and self-healing properties. The rigid bicyclic structure of ISB improved tensile properties, while the urethane bonds formed between the hydroxyl groups in ISB and isocyanate exhibited reversible characteristics at elevated temperatures, significantly enhancing the self-healing performance of DIS-based PUE compared to the control PUE (self-healing efficiency: 98% for DIS-based PUE vs. 65% for control PUE). The dynamic mechanical and rheological properties of DIS-based PUE were investigated to confirm their relationship with self-healing performance. The DIS-based PUE, featuring reversible urethane bonds, demonstrated rapid stress relaxation and maintained constant normal stress under external stimuli, contributing to its improved self-healing capabilities. Thus, ISB can be regarded as a promising bio-resource for synthesizing bio-based polyester polyols and, consequently, PUE with superior mechanical and self-healing properties.

## 1. Introduction

Polyurethanes (PUs) are versatile polymers used in a wide range of applications, from everyday products to various industries, including rigid, semi-rigid, and flexible polyurethane foams, coatings, adhesives, sealants, and elastomers. Among the various PU types, polyurethane elastomers (PUE) are widely utilized in automotive parts, artificial tissues and organs, and medical devices due to their outstanding chemical resistance, abrasion resistance, mechanical strength, and elasticity [1,2,3,4,5,6,7,8,9]. Thermoplastic PUs for elastomers are generally block copolymers formed by the addition polymerization of hydroxyl and isocyanate groups, consisting of alternating thermodynamically incompatible rigid and flexible segments, known as hard and soft segments, respectively. Furthermore, ongoing advancements in PUEs with diverse physical properties are achieved by modifying the chemical structure and types of raw materials, such as macro-diols, chain extenders, and isocyanates [10,11,12,13,14,15,16].

Increasing environmental concerns and depletion of petroleum-based resources have heightened interest in eco-friendly, bio-based materials from natural sources. Since polyols and diisocyanates, which are the major raw materials for manufacturing PUs, are based on petrochemical products, many efforts have been made to replace them with bio-based materials derived from natural or plant-based materials that are not toxic and harmful to humans [1,2,3,4,5,6,7,8,9,10,11,12,13]. One of many attempts has been to replace the polyol part occupying the largest weight fraction in PUs with bio-based polyols [1,3,4,9,13,14,15,16]. For example, Bueno-Ferre et al. reported bio-based thermoplastic polyurethanes (TPUs) prepared with dimer fatty acid-based polyol [13]. They investigated the effect of hard segment content on the morphology and physical properties of PUs. As a result, they demonstrated that the bio-based TPU can be tailored to required thermal and mechanical properties by adjusting the hard segment content. Datta et al. studied the preparation of bio-PUs based on synthetic compounds and bio-components [9]. Bio-PUs were prepared by mixing commercial poly(tetramethylene ether) glycol with soybean oil hydroxylated with bio-based 1,2- or 1,3-propanediol. The bio-PUs represented good mechanical properties and a possibility of the application in light industry. The authors emphasized the importance and advantages of biomaterials and bio-PU in terms of economic cost and the environment.

Isosorbide (ISB) is one of the bio-based materials that can be obtained via dehydration of D-sorbitol, which is also derived from D-glucose, in the presence of an acid catalyst. ISB having hydroxyl groups at positions of 2 and 5 carbons offers many possibilities in the applications of polymers [17,18,19,20,21,22]. For example, ISB can be used to manufacture polyesters through a condensation reaction with diacids [18,23,24,25], or used as a polyol or chain extender in the production of PUs [26]. Because of its unique rigid bicyclic structure as well as its non-toxicity, the incorporation of ISB into polymers imparts the improved mechanical properties and thermal stability, and even ISB can replace the role of aromatic diols due to its rigid structure and the presence of bulky hydroxyl groups [27,28].

The active hydrogen in phenolic hydroxyl groups is known to react with isocyanate groups (NCO), creating relatively weak, reversible urethane bonds. The hydroxyl groups and isocyanate groups are regenerated from the weak urethane bonds by reversible reaction at elevated temperatures (100–200 °C) [29,30,31,32]. The reversibility of the urethane bonds can be used for various applications as follows: (1) Reversible phenolic hydroxyl groups can be used as blocking agents to improve the isocyanate stability during handling and storage [29,30]. When the temperature at which the reversible reaction occurs is reached, the isocyanate groups are regenerated and participate in further reactions with hydroxyl or amine groups to form urethane or urea linkages with thermal stability. (2) The reversible feature of urethane bonds can be also utilized to create intrinsic self-healable PU coatings and films [33]. In particular, it makes the melt processing or re-molding possible at relatively low temperatures. In our recent study, we found that the urethane groups formed by the reaction of aromatic isocyanates with hydroxyl groups of ISB exhibit reversible features at elevated temperatures [34]. Thus, in this study, bio-based PUEs were prepared to investigate the self-healing performance by introducing the reversibility of ISB-based urethane bonds. At the same time, a design to increase the bio-content of PUEs was tried by preparing fully bio-based polyol containing ISB end groups. Recent studies on bio-based self-healable elastomers have reported the possibility of self-healing through mechanisms such as hydrogen bonding [15,35], metathesis of imine bonds [36,37], and the introduction of reversible covalent bonds [38,39]. While these methods provide advantages such as the ability of self-healing and recyclability at relatively low temperatures, they also present challenges related to the thermal stability of the polymers. Furthermore, research on bio-based PUE using ISB and plant oils has investigated self-healing properties through hydrogen bonding [15]; however, to the best of our knowledge, there are no studies on bio-based PUE using fully bio-based polyester with ISB-based reversible urethane linkages for self-healing. Fully bio-based polyester polyol prepared in this study was obtained by the condensation reaction of ISB with dimer fatty acid (DA). DA used as another renewable precursor in the synthesis of ISB-based polyester polyol is a bio-based resource derived from un-saturated natural oils such as oleic acid or linoleic acid [40]. DA also has branched alkyl chains, which enables the glass transition temperature reduction and fluidity of the final products. In this study, the effects of ISB-based polyester polyol (DIS) on mechanical and thermal properties as well as self-healing performance of PUEs were investigated. Furthermore, the dynamic mechanical and rheological properties of the self-healable PUEs with reversible ISB-based urethane linkages were discussed.

## 2. Materials and Methods

### 2.1. Materials

ISB (molecular weight: 146.14 g/mol) was provided from Samyang Co., Ltd. (Daejeon-si, Republic of Korea). Dimer fatty acid hydrogenated (DA, Pripol 1006) with a functionality of 2 was supplied from CRODA KOREA (Seongnam-si, Republic of Korea). Titanium (IV) *n*-butoxide (TBT) purchased from Sigma-Aldrich (Goyang-si, Republic of Korea) was used as a catalyst for esterification. 4,4-Diphenylmethane diisocyanate (MDI) and 1,4-butandiol (BD) were purchased from Sigma-Aldrich (Goyang-si, Republic of Korea) and used to prepare PUE. Priplast 3238 (molecular weight: 2000 g/mol and average functionality: 2), a fully bio-based polyester polyol based on DA (molecular weight: 2000 g/mol and functionality: 2), was supplied from CRODA (Seongnam-si, Republic of Korea) and used to prepare a control PUE without ISB.

### 2.2. Synthesis of ISB and DA Based Polyester Polyol

**Scheme 1:**
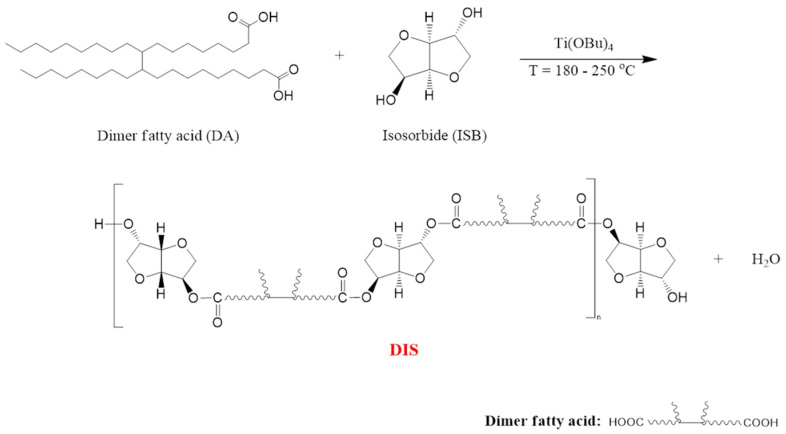
Scheme for synthesis of bio-based polyester polyol using ISB and DA.

Fully bio-based polyester polyol based on ISB and DA was synthesized via esterification reaction according to the following procedure: ISB (160.7 g, 1.10 mol) and DA (429.3 g, 0.75 mol) were weighed into a 1-L round-bottom reactor. The reactor was equipped with thermocouple, Dean-Stark condenser, and mechanical stirrer. While stirring, the reactor was heated to 180 °C under argon atmosphere, and TBT as a catalyst (0.02 mol% with respect to the DA) dissolved in toluene was added to the reaction mixture. Then, the reaction temperature was increased stepwise up to 250 °C. After collecting around 90% condensation product (water) in a Dean-Stark apparatus, the mixture further reacted under vacuum with around 2 mmHg to remove the water perfectly and to improve the yield. The esterification reaction between ISB and DA was monitored by the reduction in acid value. Subsequently, the reaction mixture was allowed to cool to room temperature, dissolved in ethyl acetate, and washed with distilled water several times. Finally, high-viscous ISB-based polyester polyol with a light yellow color was obtained and designated as DIS. The scheme for synthesizing DIS is shown in Scheme 1.

Fourier transform infrared (FTIR) spectrum (Figure S1, cm^−1^): 3457 (–OH), 2919 (sp^3^ C–H), 2850 (sp^3^ C–H), 1741 (–COO–), 1462, 1415, 1377, 1236, 1167, 1090, 1049, 1013, 979, 913, 869, 834, 774, 724, 666, 607.

^1^H nuclear magnetic resonance (NMR) spectrum (Figure S2, 600 MHz, CDCl3, δ/ppm): 0.51–1.82 (m, 193H, –C**H**–, –C**H_2_**–, and –C**H_3_** in DA), 2.34 (t, 12H, –C**H_2_**–COO–), 3.55 (m, 2H, –C**H**–), 4.45 (d, 4H, –C**H**–), 4.60 (m, 2H, –C**H**–), 4.80 (m, 2H, –O**H**), 5.17 (m, 6H, –C**H**–).

### 2.3. Preparation of PUE Based on DIS

**Scheme 2:**
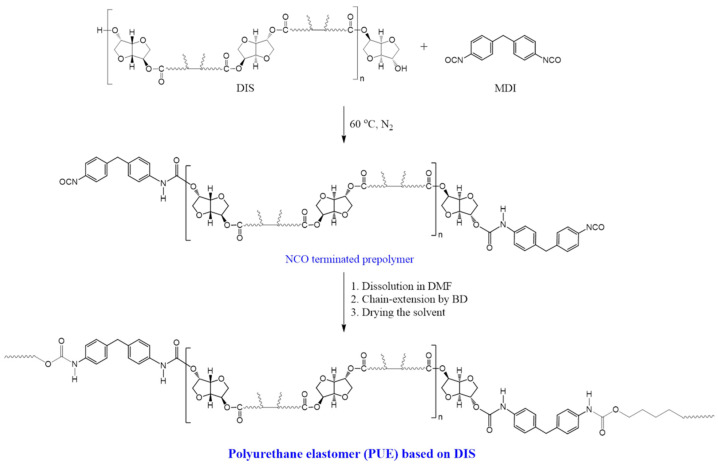
Scheme for synthetic route of DIS-PU.

PUE based on DIS was prepared by the pre-polymer method. To prepare the isocyanate-terminated pre-polymer, 1 mol of DIS and 2 mol of MDI were added to a round-bottom reactor equipped with a thermocouple, nitrogen (N_2_) inlet, and mechanical stirrer. The reaction was carried out at 70 °C and continued until reaching the theoretical isocyanate (NCO) value. NCO value was measured by the back titration method (ASTM D2572-19) [41] using 0.1 N dibutylamine solution in toluene. Subsequently, the pre-polymer was dissolved in anhydrous N,N-dimethylformamide (DMF) and polymerized by adding the pre-calculated amount of BD (1 mol) as chain extension. Then, the mixture solution was poured into an open glass mold and kept in a convection oven set at 110 °C to remove the solvent and obtain the elastomer film. PUE based on DIS was designated as DIS-PU. The synthetic route of DIS-PU is shown in Scheme 2. To confirm the effect of ISB in PUE, the control PUE was also prepared by using commercial bio-based polyester polyol based on DA (Priplaste 3238; C-PES) according to the same procedures as DIS-PU and designated as C-PU. The synthetic route of C-PU is shown in Figure S3. The composition and characteristics of DIS-PU and C-PU are listed in Table 1. The successful syntheses of both PUE (DIS-PU and C-PU) were confirmed by employing ^1^H and ^13^C NMR spectroscopy as shown in Figures S4 and S5.

### 2.4. Characterization

Bio-based polyester polyol from ISB and DA (DIS) was characterized by employing a Fourier transform infrared (FTIR) spectroscopy (FTIR 2000, JASCO) and ^1^H NMR spectrometer (600 MHz, JNM-ECA600, JEOL Ltd., Tokyo, Japan) at room temperature in CDCl_3_ as the solvent. FTIR spectrum of DIS was recorded in the wavenumber range from 4000 to 500 cm^−1^ at a resolution of 4 cm^−1^. The hydroxyl number and acid value of DIS were measured according to ASTM D4274 D [42] and ASTM D974-22 [43], respectively.

Characteristics of PUEs were investigated according to the following equipment and methods: Molecular weight of PUEs was investigated using gel permeation chromatography (GPC) (Agilent 1200S, Agilent, Santa Clara, CA, USA) with a refractive index detector (Optilab rEX, Wyatt). A sample solution dissolved in DMF/tetrahydrofuran mixture (1/1, *w*/*w*) was passed through a column calibrated to a polystyrene standard at a flow rate of 1.0 mL/min. Thermal properties of PUEs were examined by using DSC (Q20, TA instrument) and TGA (Q50, TA instrument, New Castle, DA, USA) under N_2_ atmosphere. DSC was performed ranging from −80 to 230 °C at 10 °C/min of heating rate. For TGA measurement, around 10 mg samples were loaded on the platinum pan and heated from 30 to 800 °C at 20 °C/min of heating rate. Thermo-reversible feature of urethane linkages was examined by using FTIR (FTIR 2000, JASCO, Tokyo, Japan) equipped with a heating block and thermo-controller. Dynamic mechanical properties (−100~200 °C, 5 °C/min of heating rate, 1 Hz of frequency) and stress relaxation (130, 140, 150, 160, and 170 °C and 1% strain) were investigated using a dynamic mechanical analyzer (DMA) (Q800, TA instrument). Tensile properties and self-healing performance were examined using a universal testing machine (UTM) (LR5K Plus, LLOYD, Brimingham, UK) at room temperature. Dog bone-shaped specimens were used for the measurements, and the crosshead speed was set at 250 mm/min. The tensile tests of three specimens per sample were evaluated, and the representative data were used for analysis. Scratch healing on the surface of PUE films was confirmed using a scanning electron microscope (SEM) (AIS2100C, SERON Technologies, Seoul, Republic of Korea) at an accelerating voltage of 20 kV. Before measurement, the samples were coated with gold to avoid charging of the electrons. Rheological properties of PUE melts were evaluated employing a parallel-plate rheometer (AR 2000, TA instrument, New Castle, DE, USA). The sample was placed on the 25-mm parallel plate in the heating furnace purging N_2_ gas and then heated to 180 °C (gap: 1000 µm). The steady-state flow test was performed at shear rates ranging from 0.1 to 100 s^−1,^ and the frequency sweep test was performed at angular frequencies from 628 to 0.1 rad/s at 1% strain.

## 3. Results and Discussion

### 3.1. Characteristics of DIS

Fully bio-based polyester polyol was synthesized via an esterification reaction using ISB and DA. Table 2 shows the characteristics of DIS. The molecular weight obtained by the hydroxyl value and GPC showed similar values, and it showed a good agreement with the targeting molecular weight of 2000 g/mol. The structural analysis was carried out using FTIR (Figure S1), ^1^H NMR (Figure S2), and ^13^C NMR (Figure S6) spectroscopy. In the FTIR spectrum of DIS (Figure S1), the carboxylic acid (–COOH) functional group disappeared almost perfectly (1710 cm^−1^), and new absorption peaks corresponding to hydroxyl (–OH) and carbonyl ester (–COO–) functional groups were observed at 3457 and 1741 cm^−1^, resulting from the esterification reaction between ISB and DA. The chemical structure of DIS was also confirmed using ^1^H NMR (Figure S2). ^1^H NMR spectrum of DIS showed reasonable assignments of signals and good agreement with its chemical structure. The signals ranging from 0.51 to 1.82 ppm represented the aliphatic methylene protons of DA [44]. The detail assignment of DA was presented in Figure S7. The successful synthesis of DIS was also confirmed by ^13^C NMR as shown in Figure S6. The chemical shift of carboxylic acid appearing at 180 ppm was shifted to 173 ppm, indicating the formation of the ester functional group. In general, ISB-based polyester polyols prepared from aliphatic diacids, i.e., succinic acid or butanedioic acid, have a high glass transition temperature above 50 °C [24,45]. It makes a polyester polyol to lose the fluidity. On the other hand, DA is derived from the dimerization of fatty acids with long aliphatic chains. Compared to the previously mentioned aliphatic diacid, DA exhibits weaker intermolecular interactions and a more flexible molecular chain, allowing for better mobility. For this reason, the ISB-based polyester polyol using DA exhibited a relatively low glass transition temperature. Thus, in this work, DA was used as a diacid to obtain the fully bio-based polyester polyol having fluidity. As a result, high-viscous polyester polyol with a low glass transition temperature (−27.7 °C) was obtained (Table 2). DIS also exhibited a single glass transition temperature (T_g_) and thermal decomposition temperature (T_d_). It means that the esterification reaction was successfully performed and DIS did not contain any impurities, such as low molecular products or raw materials. DSC and TGA thermograms (thermogravimetry (TG) and derivative TG (DTG)) are presented in Figures S8 and S9.

### 3.2. Characteristics of PUE Based on DIS

To confirm the effect of ISB in DIS on the thermal and mechanical properties as well as self-healing performance, control PUE was prepared from DA-based polyester polyol with aliphatic hydroxyl groups and the same molecular weight as DIS. Furthermore, in the preparation of PUE, DIS-PU and C-PU were prepared to constitute a similar chemical composition and hard segment content. The composition and characteristics of both PUEs are listed in Table 1. The number of molecular weights of DIS-PU was slightly lower than that of C-PU, which was attributed to the lower reactivity of bulky hydroxyl groups in DIS and the high viscosity of the pre-polymer based on DIS. When fully bio-based polyester polyol was incorporated into PUE, the bio content (by weight) was quite high (Table 1) due to the large weight fraction of polyol in the manufacture of PUE.

**Figure 1:**
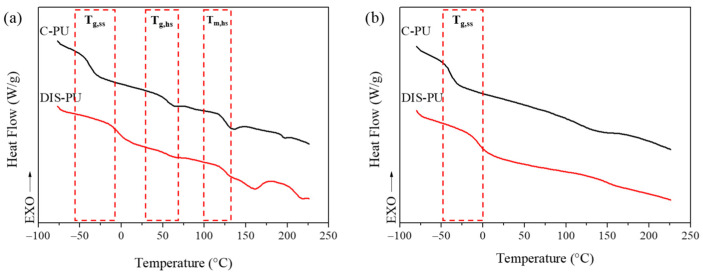
DSC thermograms of DIS-PU and C-PU: (**a**) first heating scan; (**b**) second heating scan.

Figure 1 shows DSC thermograms of DIS-PU and C-PU and Table S1 lists their characteristic endothermic peaks. T_g_ of the soft segment (T_g,ss_) of DIS-PU showed a higher value at −15.9 °C compared with that of C-PU (−44.7 °C) due to the stiff bicyclic structure of ISB. It may lead to a restriction of the molecular chain mobility, which resulted in an increase in T_g,ss_. The endothermic peaks at around 50 °C and 117 °C corresponded to the T_g_ and T_m_ of the hard segment (T_g,hs_ and T_m,hs_), respectively. These characteristic endotherms did not show significant difference to C-PU. However, both PUEs exhibited relatively low T_m,hs_ compared to typical PUE (T_m,hs_ = 150; 200 °C) synthesized from polyether diol (poly(tetrahydrofuran)), MDI, and BD as a chain extender [46].

PUE is a segmented block copolymer consisting of the soft domain (polyol segment) and the hard domain (urethane segment), and hard segments are packed by physical crosslinking through hydrogen bonding between urethane linkages. In this study, the aliphatic fatty acid chain in DA may hinder the hydrogen bonding between the hard segments, resulting in the weakening of its association. As a result, the hard segments of DIS-PU and C-PU were molten at relatively low temperatures. Figure S10 shows FTIR spectra of DIS-PU and C-PU. In the FTIR spectrum of typical PUE, the peaks appearing at 1736 cm^−1^ and 1703 cm^−1^ were assigned to the free urethane carbonyl group and hydrogen-bonded carbonyl group by association of hard segments [47,48]. As expected, the peak intensity of the free carbonyl group (1736 cm^−1^) is much more dominant than that of the hydrogen-bonded carbonyl group (1703 cm^−1^), supporting the lower T_m,hs_ of DIS-PU and C-PU.

**Figure 2:**
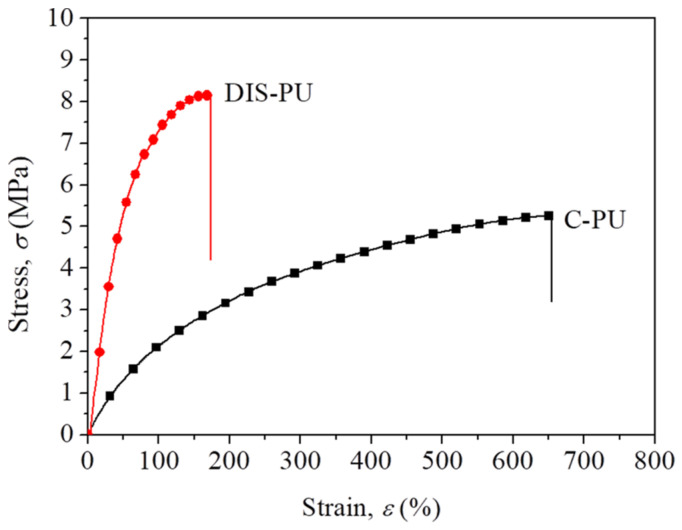
Stress-strain curves of DIS-PU and C-PU.

Figure 2 shows the stress-strain curves of DIS-PU and C-PU. The tensile strength (*σ*) and Young’s modulus (*E*) of DIS-PU exhibited significant improvement compared with those of C-PU due to the incorporation of DIS with a stiff bicyclic structure. For example, *σ* and *E* of DIS-PU were 8.2 MPa and 18.4 MPa, while those of C-PU were 5.3 MPa and 3.4 MPa, respectively. The *σ* and *E* of DIS-PU were improved by approximately 55% and 448% compared with C-PU. Accordingly, the elongation at break (*ε*) was significantly decreased from 654.6% for C-PU to 173.2% for DIS-PU. It reveals that eco-friendly and hardy PUE can be obtained by incorporation of ISB-based.

**Figure 3:**
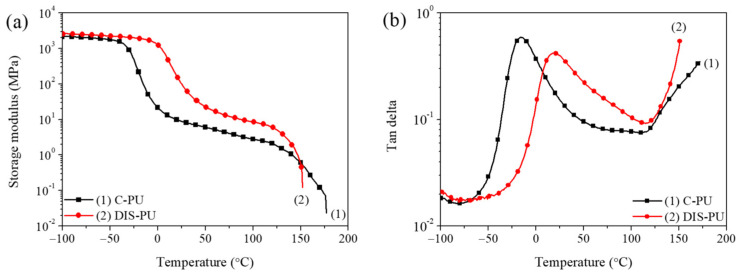
Temperature-dependent (**a**) storage modulus and (**b**) tan delta curves of DIS-PU and C-PU.

Figure 3 shows the storage modulus and tan delta (tan δ) curves of DIS-PU and C-PU obtained by DMA measurement. Both PUEs showed an obvious phase transition from glassy to rubbery with increasing temperature. As confirmed in the DSC result (see Figure 1), DIS-PU showed a glass-rubber transition at higher temperatures compared with C-PU. T_g_ was also determined by the maximum point in tan δ curves and summarized in Table S1. The result showed a good agreement with DSC results, namely the higher T_g_ was observed for DIS-PU than C-PU (T_g_ of DIS-PU and C-PU was 19.5 and −15.6 °C). Furthermore, DIS-PU exhibited a higher storage modulus in the overall temperature range compared with C-PU due to the high rigidity of ISB that makes up DIS. Interestingly, after the rubbery plateau region, the storage modulus of DIS-PU sharply dropped at the lower temperature compared with C-PU (melting temperature (T_m_) of DIS-PU and C-PU was 152 and 177 °C, respectively). It can be attributed to the reversible feature of urethane linkages formed by the reaction between the hydroxyl groups in ISB and NCO groups as reported by Kim et al. [49].

**Figure 4:**
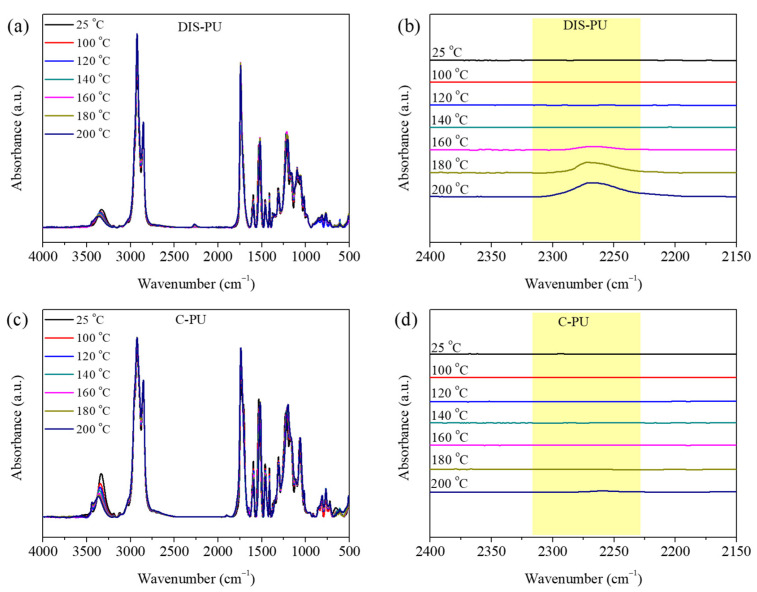
Temperature-dependent FTIR spectra of (**a**,**b**) DIS-PU and (**c**,**d**) C-PU; (**b**) DIS-PU expanded at 2150–2400 cm^−1^; (**d**) C-PU expanded at 2150–2400 cm^−1^.

The thermo-reversible feature of ISB-based urethane linkages was investigated by confirming re-generated NCO groups at elevated temperatures using FTIR spectroscopy [33]. Figure 4 shows the FTIR spectra of DIS-PU and C-PU at different temperatures. The FTIR spectra obtained were normalized against the intensity of the absorption peak assigned to *sp^3^* C–H (2850 cm^−1^) to confirm the temperature-dependent changes. In general, the absorption peak of the NCO functional group can be observed at 2270 cm^−1^. C-PU did not show the re-generated NCO group across the overall temperature range, while the DIS-PU containing ISB-based urethane linkages showed a clear absorption peak of the re-generated NCO group at 2270 cm^−1^, as the temperature was higher than 160 °C. This result is very close to the T_m_ (152 °C) observed in the storage modulus curve of DIS-PU. Consequently, the reversibility of ISB-based urethane linkages was confirmed at elevated temperatures higher than 160 °C, allowing for intermolecular rearrangement. It implies that self-healing performance of DIS-PU can be implemented at this temperature through the reversibility of ISB-based urethane linkages.

**Figure 5:**
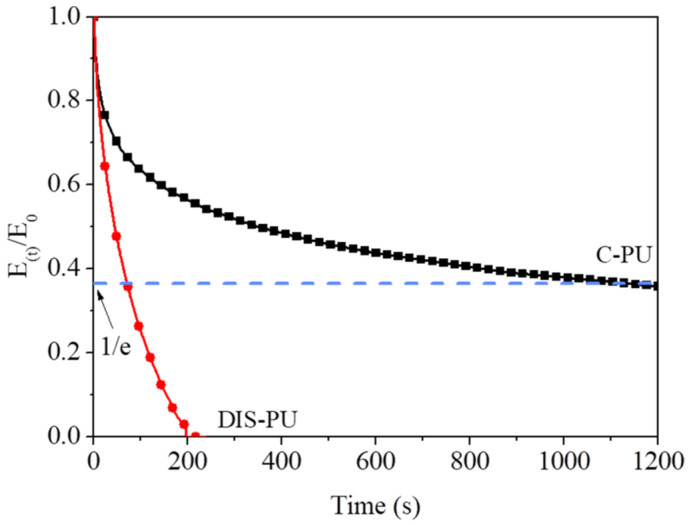
Normalized stress relaxation modulus of DIS-PU and C-PU at 160 °C (the blue dot line indicates the 1/e from the initial value).

Stress relaxation is a phenomenon where stress decreases with time with a response of stress to the constant strain, which is one of the important parameters for self-healing polymers [50,51,52]. The stress relaxation time (*τ*(t)) is a time at which the initial modulus value reaches 1/e (0.37) in normalized relaxation modulus, which is an indicator of the rate of stress relaxation. That is, the shorter *τ*(t), the faster the stress relaxation occurs and the stress decreases rapidly. The normalized relaxation modulus (E_(t)_/E_0_) of DIS-PU and C-PU at 160 °C is shown in Figure 5. In general, stress relaxation of viscoelastic polymers can be induced by chain mobility and loosening of entangled chains [53,54]. Thus, both PUEs showed the stress relaxation behavior with increasing time, and the relaxation modulus dropped with increasing temperature. E_(t)_/E_0_ and relaxation modulus of DIS-PU or C-PU at different temperatures are given in Figure S11 and Figure S12, respectively. It was also found that the normalization relaxation modulus of DIS-PU was dropped sharply, reaching 1/e faster compared with C-PU. The *τ*(t) of DIS-PU and C-PU were 69 and 1089 s at 160 °C, respectively (*τ*(t) at different temperatures of DIS-PU and C-PU are summarized in Table S2). In addition, the E_(t)_/E_0_ and relaxation modulus of C-PU decreased slightly with increasing temperature, while DIS-PU decreased rapidly. In particular, at around the temperature where the reversibility of ISB-based urethane bonds was observed (150–160 °C), the relaxation modulus of DIS-PU dropped more rapidly, reaching a faster *τ*(t). Namely, the fast *τ*(t) of DIS-PU is due to the reversibility of ISB-based urethane bonds under the measured conditions. Therefore, PUEs with ISB-based urethane bonds (DIS-PU) can be expected to exhibit faster and better self-healing performance as compared to C-PU.

**Figure 6:**
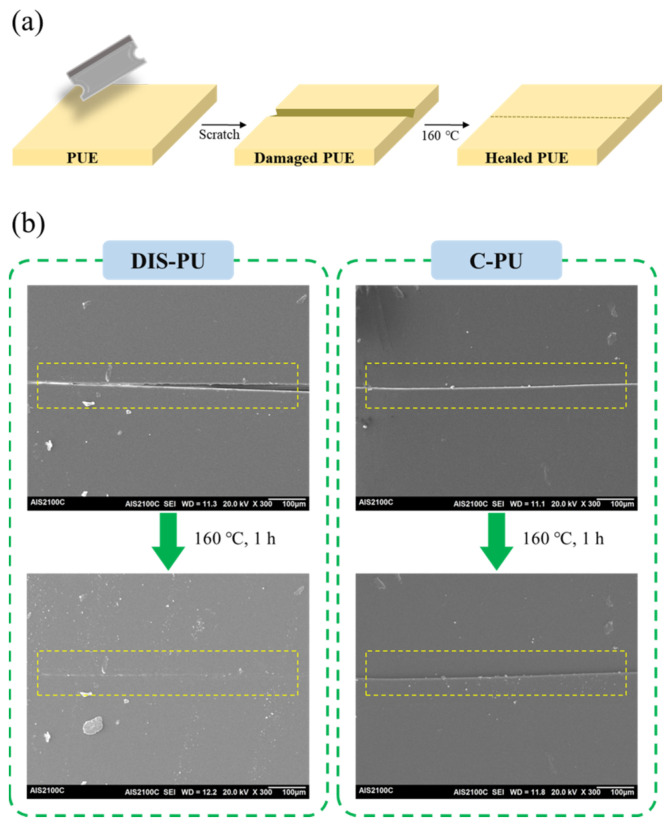
(**a**) Schematic illustration for scratch healing of PUEs and (**b**) SEM images of damaged and self-healed DIS-PU and C-PU (self-healing was performed at 160 °C for 1 h).

**Figure 7:**
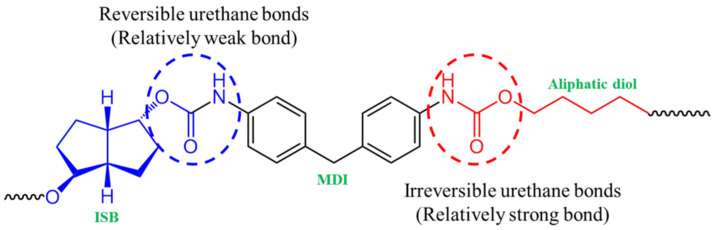
Structural illustration for the reversible feature of ISB-based urethane bonds.

SEM was used to evaluate the self-healing performance of DIS-PU. The surface of DIS-PU was scratched using a razor blade and allowed to be healed at 160 °C for 1 h (see Figure 6a). SEM images of DIS-PU and C-PU for self-healing are shown in Figure 6b. The scratch on the surface of DIS-PU almost disappeared and healed, attributable to the reversible reaction of ISB-based urethane linkages (relatively weak bond). On the other hand, the scratch on the surface of C-PU remained almost intact due to the irreversibility of urethane bonds formed by the reaction of NCO groups with aliphatic hydroxyl groups (relatively strong bond) (see Figure 7).

**Figure 8:**
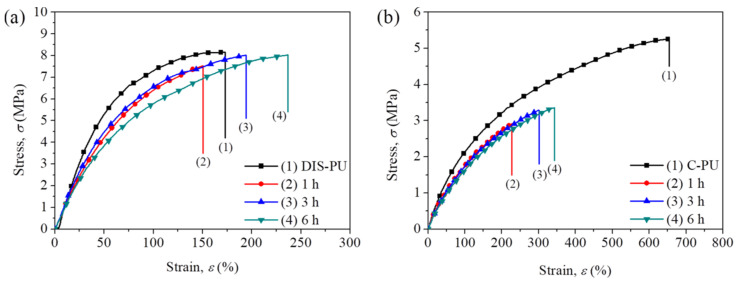
Stress-strain curves of the original specimens and self-healed specimens at different times (1 h, 3 h, and 6 h) (healing temperature: 160 °C): (**a**) DIS-PU and (**b**) C-PU.

**Figure 9:**
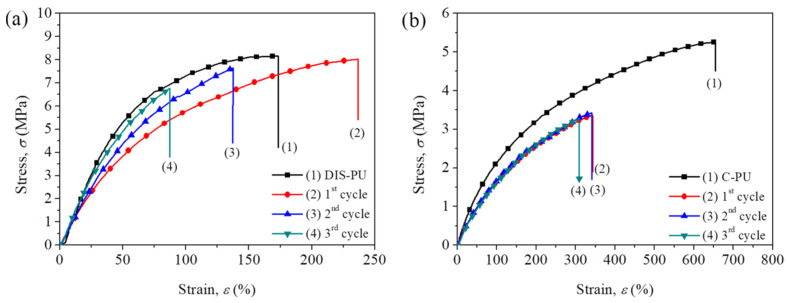
Stress-strain curves of the original specimens and self-healed specimens at different numbers of cutting and healing cycles (healing was carried out at 160 °C for 6 h: (**a**) DIS-PU and (**b**) C-PU.

Self-healing properties of DIS-PU were also evaluated using the cutting and healing method. The dog bone-shaped specimens of DIS-PU and C-PU were prepared to measure the original tensile properties (Figure S13a). Then, the center of the specimens were cut, and the two pieces were brought in close contact with each other (Figure S13b). The cut specimens were treated at 160 °C to allow self-healing (Figure S13c). The quantitative analysis on self-healing properties of DIS-PU and C-PU was carried out by calculating the healing efficiency (%) after the tensile test of the healed samples. Self-healing efficiency was calculated using the following Equation (1):

Self-healing efficiency (%) = *σ*_healed_/*σ*_original_ × 100% (1)

where *σ*_original_ and *σ*_healed_ are the tensile strengths of original specimens (before self-healing) and self-healed specimens, respectively. The stress-strain curves of healed DIS-PU and C-PU depending on different healing times (1, 3, and 6 h) are given in Figure 8. The self-healing efficiencies of healed DIS-PU and C-PU are summarized in Table 3 and the results of the tensile strength and elongation at break of both PUE are summarized in Table S3. It was found that the self-healing efficiencies for the tensile strength of both DIS-PU and C-PU increased with increasing healing time. The self-healing efficiency of DIS-PU was improved to 98.5% after 6 h of healing time, while C-PU exhibited a lower self-healing efficiency compared with DIS-PU (63.8% after 6 h of healing time). Y. Lin et al. developed a self-healable bio-based PU utilizing reversible urethane linkages derived from a polyphenol [55]. The self-healing efficiencies were 73.5% for tensile strengths and 84.3% for elongation at break at 120 °C. The bio-based PUE synthesized by Xu et al. achieved a remarkable self-healing efficiency exceeding 95.0% of its tensile strength at 120 °C by incorporating the combination of reversible urethane units and metathesis of imine bonds [56]. Another study conducted by Lee et al. demonstrated that bio-based PUE, which incorporates dimerized eugenol and reversible phenolic urethane linkages, exhibited a self-healing efficiency exceeding 99.0% at 150 °C after a duration of 3 h [57]. Although the DIS-PU performed self-healing at a relatively high temperature (160 °C), it still exhibited self-healing efficiency comparable to that of other bio-based elastomers. In the experiment of repeated cycles of cutting and healing as shown in Figure 9 and Table 4 (also in Table S4), the self-healing efficiency of DIS-PU was slightly reduced with increasing the number of repeated cycles but still exhibited a relatively high self-healing efficiency of over 80% (self-healing efficiency of 3rd cycle was 83.0%). On the other hand, C-PU did not show a significant change in self-healing efficiency as the repeated cycles of cutting and healing increased. Although self-healing efficiency of C-PU was lower than that of DIS-PU, it showed almost constant self-healing efficiency of 62–65% in repeated experiments up to three times. This is due to the fact that the C-PU with long fatty acid alkyl chains can be healed by intermolecular parameters, such as chain diffusion, entanglement, and rearrangement above T_g_ [58,59]. Thus, it demonstrates that the self-healing efficiency of PUEs can be improved by the introduction of dynamic covalent bonds, i.e., thermally reversible ISB-based urethane bonds studied in this work. The self-healing efficiencies (%) for the elongation at break of DIS-PU and C-PU were also evaluated using Equation (1), with the results presented in Table 3 and Table 4. Both the DIS-PU and C-PU exhibited increased self-healing efficiency as the healing time increased. Interestingly, DIS-PU demonstrated better self-healing performance compared to C-PU, being attributed to the introduction of reversible ISB-based urethane bonds. The self-healing efficiencies for the elongation at break of DIS-PU were 86.8%, 112.1%, and 136.7% for the healing times of 1, 3, and 6 h, respectively, while those of C-PU were 34.8%, 45.9%, and 52.5%. Although DIS-PU exhibited higher self-healing efficiencies for the elongation at break than C-PU, the self-healing efficiency of both PUE tended to decrease with an increase in the number of cycles of cutting and healing. It is speculated that the strain generally occurs as the entangled polymer chain is released by tensile force, which can be observed in the soft segment of the PU. However, since the soft segment corresponding to DA does not have any self-healable functional groups, this caused a decrease in the self-healing efficiency for the elongation at break during repeated tests of cutting and healing. In addition, no changes in molecular structure and molecular weight were observed after self-healing of both PUEs (DIS-PU and C-PU) at 160 °C for 6 h, which were confirmed using FTIR and GPC (see Figure S14 and Table S5). DIS-PU and C-PU were also thermally stable, namely, the weight loss did not occur up to 260 °C in the TGA curves of both the PUEs (see Figure S15). It reveals that there are no concerns about the side reaction or thermal decomposition of PUEs during the self-healing at 160 °C.

**Figure 10:**
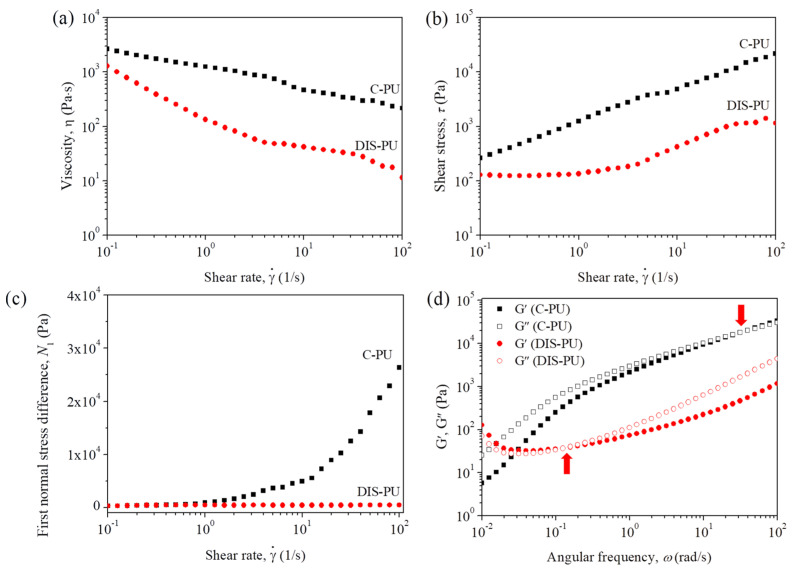
Rheological properties of DIS-PU and C-PU at 180 °C: (**a**) shear viscosity, (**b**) shear stress, (**c**) first normal stress difference versus shear rate, and (**d**) G′ (closed) and G″ (opened) versus angular frequency.

The steady-state flow and frequency sweep tests of both PUE melts were carried out at 180 °C to investigate the effects of reversible ISB-based urethane linkages on the rheological properties in a molten state. The rheological properties under shear or oscillation are shown in Figure 10.

The shear viscosity (η) of both PUEs showed non-Newtonian behaviors with increasing shear rate due mainly to the loosening of the entangled polymer chains under the shear (Figure 10a) [54,60]. DIS-PU melts exhibited lower and faster decrease in melt viscosity than C-PU over the whole shear rate. It may be attributed to the low average molecular weight of DIS-PU compared to C-PU, as shown in Table 1. Interestingly, it was found that the plateau shear stress of DIS-PU was observed at low shear rate; namely, DIS-PU with reversible ISB-based urethane linkages exhibited yield behavior. When plotting shear viscosity versus shear stress, the yield behavior of DIS-PU could be obviously confirmed (see Figure S16). It can be explained that the dissociation of ISB-based urethane linkages occurred in the molecules with relatively low molecular weight under shear, and it makes the polymer melts flow more easily, which resulted in a more rapid decrease in shear viscosity than C-PU after yield stress. It means that the reversibility of ISB-based urethane linkages can induce the easier melt processing of PUEs by significantly reducing the melt viscosity above the yield stress. We also plotted the first normal stress difference (*N*_1_) versus shear rate in Figure 10c. In general, *N*_1_ of oils, polymer solutions, suspensions, and polymer fluids tend to increase with increasing shear rate [61,62,63]. C-PU showed a typical *N*_1_ increase with increasing shear rate. On the other hand, DIS-PU showed almost constant *N*_1_ with increasing shear rate. It can be inferred that the reversibility of ISB-based urethane bonds may dissipate the normal stress formed in the polymer melt during shearing. In accordance with this, it was also found that the shear stress (*τ*) as a function of shear rate of DIS-PU melt was smaller than that of C-PU (see Figure 10b). Thus, the stress dissipation by the reversibility of ISB-based urethane bonds will alleviate the expansion of polymeric fluids such as die-swell in the practical processing of polymers [64].

The plots of storage modulus (G′) and loss modulus (G″) versus angular frequency are presented in Figure 10d (the plots of time-dependent G′ and G″ were given in Figure S17). Crossovers of G′ and G″ of DIS-PU and C-PU melts were observed at different ω (DIS-PU: 0.40 rad/s and C-PU: 31.5 rad/s). The crossover of G′ and G″ of the C-PU melt was observed at a relatively high ω (relatively early time), which is attributed to the disentanglement of molecular chains under oscillation. In addition, the C-PU melt exhibited liquid-like behavior (G″ > G′) at low ω after the crossover. On the other hand, DIS-PU melt showed liquid-like behavior at high ω, and the crossover of G′ and G″ was observed at relatively low ω to form a gel-like structure (G′ > G″). It may be due to the association of the free hydroxyl or NCO functional groups formed by the reversible reaction of ISB-based urethane linkages. The low ω can be considered to be in a static state with hardly any motions compared with high ω, relatively. Thus, the dissociated molecules can approach each other and partially form the transient reversible associations. Another reason for the crossover of G′ and G″ of DIS-PU is because of the formation of yield stress at low strain as confirmed in the steady-state flow test. It means that DIS-PU melt would flow above the specific strain or yield stress, and, before critical yield stress, the elasticity (G′) of polymers was predominant than viscosity (G″). The complex viscosity (η*) versus ω of DIS-PU and C-PU melts are presented in Figure S18. Both the PUE melts exhibited non-Newtonian flow; η* tended to decrease with increasing ω. η* of the C-PU melt showed the inflection point at 0.1 rad/s, which was attributed to the slope transition of G′ and G″. DIS-PU exhibited the higher η* than C-PU at low ω due to the formation of a transient gel-like structure; however, η* was sharply decreased with increasing ω attributable to the reversible reaction of ISB-based urethane linkages.

## 4. Conclusions

In this study, fully bio-based polyester polyol based on ISB and DA (DIS) was synthesized via an esterification reaction and characterized. The DIS with bulk hydroxyl end groups was used to prepare bio-based PUE with self-healing properties. The resulting DIS-PU demonstrated significantly improved Young’s modulus (18.37 MPa) and tensile strength (8.2 MPa) compared with C-PU (Young’s modulus 3.35 MPa and tensile strength 5.3 MPa for C-PU). It was attributed to the presence of ISB molecules with a rigid bicyclic structure in DIS. As the rigidity increased, the elongation at the break of DIS-PU (173.2%) was significantly lower than that of C-PU (654.6%). The self-healing performance of DIS-PU is driven by the reversible nature of ISB-based urethane linkages at elevated temperatures. FTIR spectroscopy confirmed the regeneration of NCO functional groups in DIS-PU above 160 °C, with a rapid decrease in modulus observed around 150 °C. Additionally, DIS-PU exhibited a faster stress relaxation time compared to C-PU at 160 °C, primarily due to the reversible reaction of ISB-based urethane linkages. Consequently, it was found that DIS-PU exhibited excellent self-healing performance compared to C-PU in the investigations of the scratch healing and cutting and healing tests (self-healing efficiency: 98% for DIS-PU and 64% for C-PU). In a rheological investigation of PUE melts, the lower *η* and *τ* depending on $\dot{\gamma }$ of DIS-PU were observed compared with C-PU; even DIS-PU melt exhibited yield behavior. This was due to the relatively low average molecular weight as well as the reversibility of ISB-based urethane bonds. In addition, *N*_1_ of C-PU increased significantly with increasing $\dot{\gamma }$, while that of DIS-PU was almost maintained constant due to the stress dissipation by the reversible reaction of ISB-based urethane bonds. In conclusion, ISB presents a promising and attractive bio-precursor for synthesizing bio-based polyester polyols and their corresponding PUEs, which exhibit superior mechanical properties and self-healing performance due to the reversible reactions of ISB-based urethane bonds. This study clearly indicates that achieving self-healing properties through the reversibility of ISB-based urethane linkages requires temperature to be raised above 160 °C. To address this limitation, further research is necessary to explore dynamic covalent bonds or intermolecular interactions that facilitate self-healing at lower temperatures.

## Figures and Tables

**Table 1:** Composition and characteristics of DIS-PU and C-PU.

Sample	Composition (Mole)				HC ^a^ (%)	Bio-Content ^b^ (wt.%)	Mn ^c^ (g/mol)	PDI ^d^
	DIS	C-PES	MDI	BD				
DIS-PU	1	-	2	1	22.3	77.7	13,420	3.47
C-PU	-	1	2	1	22.8	77.2	17,290	2.74

^a^ Hard segment content (HC) = W_h_/W_t_ × 100% (where W_h_ is the sum of grams of MDI and BD and W_t_ is the sum of the weight of all the raw materials for DIS-PU or C-PU); ^b^ concentration of biomass-based components; ^c^ the number average molecular weight obtained by GPC measurement; ^d^ the polydispersity index calculated as Mw/Mn (where Mw is the weight average molecular weight).

**Table 2:** Characteristics of bio-based polyester polyol based on ISB and DA.

Sample	OHV ^a^ (mg KOH/g)	AV ^b^ (mg KOH/g)	Viscosity ^c^ (Pa∙s)	T_g_^d^ (°C)	T_d_^e^ (°C)	M ^f^ (g/mol)	M_n_^g^ (g/mol)
DIS	54.5	2.80	248	−27.7	446	2058	2273
C-PES	56.4	0.30	16	−60.1	-	1989	2020

^a^ Hydroxyl value measured by titration method (ASTM D-4274 D); ^b^ Acid value measured by titration method (ASTM D974-22); ^c^ Viscosity was determined at shear rate 1 s^−1^ at 25 °C employing a parallel-plate rheometer; ^d^ Glass transition temperature determined by DSC; ^e^ Onset decomposition temperature obtained by TGA; ^f^ Molecular weight calculated by using OHV (Molecular weight = 56,100 × functionality (*f* = 2)/OHV); ^g^ The number of molecular weight obtained by GPC.

**Table 3:** Self-healing efficiencies (%) of DIS-PU and C-PU depending on the healing time.

Sample		Healing Efficiency (%)		
		1 h	3 h	6 h
Tensile Strength	DIS-PU	92.3	98.3	98.5
	C-PU	55.6	62.6	63.8
Elongation at break	DIS-PU	86.8	112.1	136.7
	C-PU	34.8	45.9	54.5

**Table 4:** Self-healing efficiencies (%) of DIS-PU and C-PU depending on the repeated cycle.

Sample		Healing Efficiency (%)		
		1st Cycle	2nd Cycle	3rd Cycle
Tensile Strength	DIS-PU	98.5	93.7	83.0
	C-PU	63.8	65.2	62.3
Elongation at break	DIS-PU	136.7	79.4	50.5
	C-PU	52.5	52.1	47.3

## Data Availability

Data are contained within the article and Supplementary Materials.

## Supplementary Materials

The following supporting information can be downloaded at: https://www.mdpi.com/article/10.3390/polym16243571/s1, Figure S1. FTIR spectra of ISB, DA, and DIS; Figure S2. ^1^H NMR spectrum of fully bio-based polyester polyol based on ISB and DA (DIS); Figure S3. Synthetic route of PUE based on C-PES (Priplast 3238) (C-PU); Figure S4. ^1^H NMR spectra of DIS-PU and C-PU; Figure S5. ^13^C NMR spectra of DIS-PU and C-PU; Figure S6. ^13^C NMR of ISB, DA, and DIS; Figure S7. ^1^H NMR spectrum of DA in CDCl_3_; Figure S8. DSC thermogram of DIS under N_2_ atmosphere; Figure S9. (a) TG and (d) DTG thermograms of ISB, DA, and DIS at N_2_ atmosphere; Table S1. Characteristic temperatures of DIS-PU and C-PU determined by DSC and DMA; Figure S10. FTIR spectra of DIS-PU and C-PU measured employing ATR mode; Figure S11. Normalized relaxation modulus (E_(t)_/E_0_) of (a) DIS-PU and (b) C-PU at different temperature; Figure S12. Stress relaxation modulus of DIS-PU and C-PU at different temperature; Table S2. Stress relaxation time (τ(t)) of DIS-PU and C-PU at different temperature; Table S3. Tensile strength and elongation at break of DIS-PU and C-PU at different healing time; Table S4. Tensile strength and elongation at break of DIS-PU and C-PU at different repeated cycles of cutting and healing; Figure S13. Photographs of dog-bone-shaped specimens of DIS-PU and C-PU and route for cutting and healing test to obtain self-healing efficiency; Figure S14. Comparisons of FTIR spectra of both DIS-PU and C-PU before and after self-healing at 160 °C for 6 h; Table S5. The number of molecular weight (M_n_) and polydispersity index (PDI) of DIS-PU and C-PU after self-healing at 160 °C; Figure S15. TG and DTG thermograms of (a) DIS-PU and (b) C-PU at N_2_ atmosphere; Table S6. Characteristic decomposition temperatures of DIS-PU and C-PU; Figure S16. Shear viscosity versus shear rate of DIS-PU and C-PU at 180 °C; Figure S17. Storage modulus (G′) and loss modulus (G″) as a function of time of DIS-PU and C-PU at 180 °C; Figure S18. Complex viscosity (η*) versus angular frequency of DIS-PU and C-PU at 180 °C.

## Abbreviations

ISB: isosorbide; DA, dimer fatty acid; DIS, ISB and DA-based polyester polyol; PUE, polyurethane elastomer; PU, polyurethane; NCO, isocyanate; MDI, 4,4-diphenylmethane diisocyanate; BD, 1,4-butanediol; TBT, titanium (IV) *n*-butoxide; DIS-PU, polyurethane elastomer based on DIS; C-PU, control PUE; FTIR, Fourier transform infrared; NMR, nuclear magnetic resonance; DMF, N,N-dimethylformamide; C-PES, control polyester polyol; HC, hard segment content; M_n_, average number of molecular weight; PDI, polydispersity index; DSC, differential scanning calorimeter; TGA, thermogravimetric analysis; GPC, gel permeation chromatography; DMA, dynamic mechanical analyzer, UTM, universal testing machine; SEM, scanning electron microscope; T_g_, glass transition temperature; T_d_, thermal decomposition temperature; TG, thermogravimetry; DTG, derivative TG; OHV, hydroxyl value; AV, acid value.
