# Supplementary material for: Thermally Healable Polyurethane Elastomers Based on Biomass Polyester Polyol from Isosorbide and Dimer Fatty Acid
Source: Polymers (Basel). 2024 Dec 20;16(24):3571. doi: 10.3390/polym16243571 (PMC11679297; doi:10.3390/polym16243571)
Supplement: Supplementary file 1 [file polymers-16-03571-s001.zip › polymers-3342276-supplementary.pdf]

Supporting Information

Thermally Healable Polyurethane Elastomers Based on Bio-mass Polyester Polyol from Isosorbide and Dimer Fatty Acid

Se-Ra Shin[1,*] *and Dai-Soo Lee*[2]

[1] Research Institute, Jungwoo Fine Co., Ltd., #63-8, 75, Seogam-ro 1-gil, Iksan-si, Jeonbuk-do, 54586, Republic of Korea; srshin1@naver.com

[2] C & S Partner, B-402, Hanam Technovalley U1 Center, Hanam city, Gyeonggi-do, 12982, Republic of Korea; dslee@jbnu.ac.kr

* To whom correspondence should be addressed: srshin1@naver.com

List of Tables

Table S1. Characteristic temperatures of DIS-PU and C-PU determined by DSC and DMA....... 7
Table S2. Stress relaxation time (τ(t)) of DIS-PU and C-PU at different temperature 9
Table S3. Tensile strength and Elongation at break of DIS-PU and C-PU at different healing time 9
Table S4. Tensile strength and Elongation at break of DIS-PU and C-PU at different repeated cycles of cutting and healing........ 10
Table S5. The number of molecular weight (M_n) and polydispersity index (PDI) of DIS-PU and C-PU after self-healing at 160 °C 11
Table S6. Characteristic decomposition temperatures of DIS-PU and C-PU 12

List of Figures

Figure S1. FTIR spectra of ISB, DA, and DIS. 3
Figure S2. ^{1}H NMR spectrum of fully biobased polyester polyol based on ISB and DA (DIS). .. 3
Figure S3. Synthetic route of PUE based on C-PES (Priplast 3238) (C-PU) 4
Figure S4. ^{1}H NMR spectra of DIS-PU and C-PU. 4
Figure S5. ^{13}C NMR spectra of DIS-PU and C-PU. 5
Figure S6. ^{13}C NMR of ISB, DA, and DIS. 5
Figure S7. ^{1}H NMR spectrum of DA in $CDCl_3$. 6
Figure S8. DSC thermogram of DIS under N_2 atmosphere. 6
Figure S9. (a) TG and (d) DTG thermograms of ISB, DA, DIS at N_2 atmosphere. 7
Figure S10. FTIR spectra of DIS-PU and C-PU measured employing ATR mode........ 8
Figure S11. Normalized relaxation modulus ($E_{(t)}/E_0$) of (a) DIS-PU and (b) C-PU at different temperature. 8
Figure S12. Stress relaxation modulus of DIS-PU and C-PU at different temperature........ 9
Figure S13. Photographs of dog-bone-shaped specimens of DIS-PU and C-PU and route for cutting & healing test to obtain self-healing efficiency. 10
Figure S14. Comparisons of FTIR spectra of both DIS-PU and C-PU before and after self-healing at 160 °C for 6 h. 11
Figure S15. TG and DTG thermograms of (a) DIS-PU and (b) C-PU at N_2 atmosphere. 12
Figure S16. Shear viscosity versus shear rate of DIS-PU and C-PU at 180 °C. 13
Figure S17. Storage modulus (G′) and loss modulus (G″) as a function of time of DIS-PU and C-PU at 180 °C. 13
Figure S18. Complex viscosity (η*) versus angular frequency of DIS-PU and C-PU at 180 °C. 14

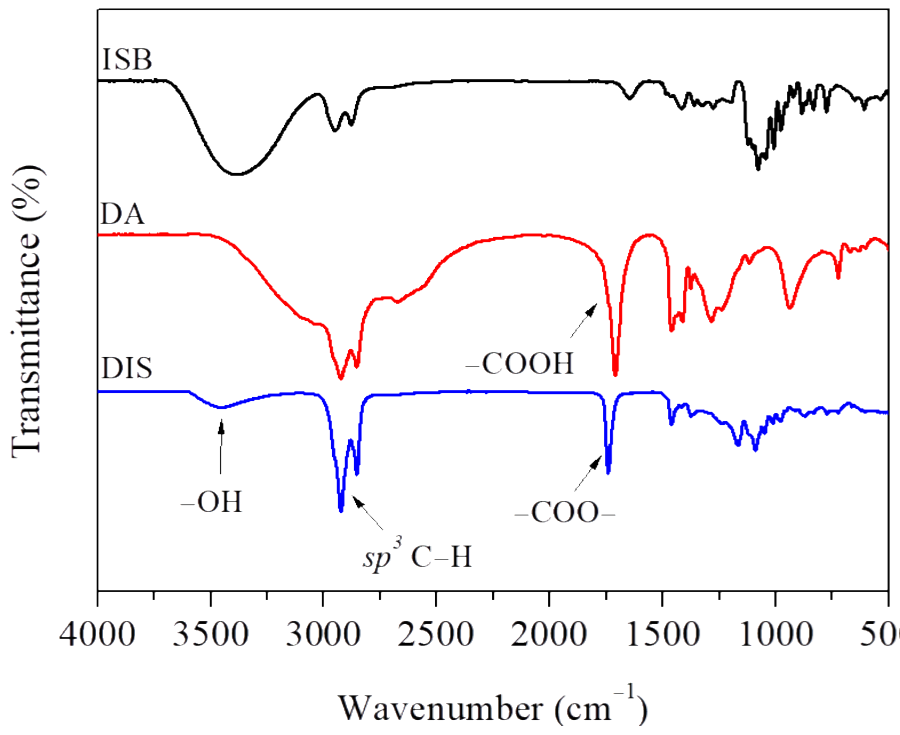

Figure S1. FTIR spectra of ISB, DA, and DIS.

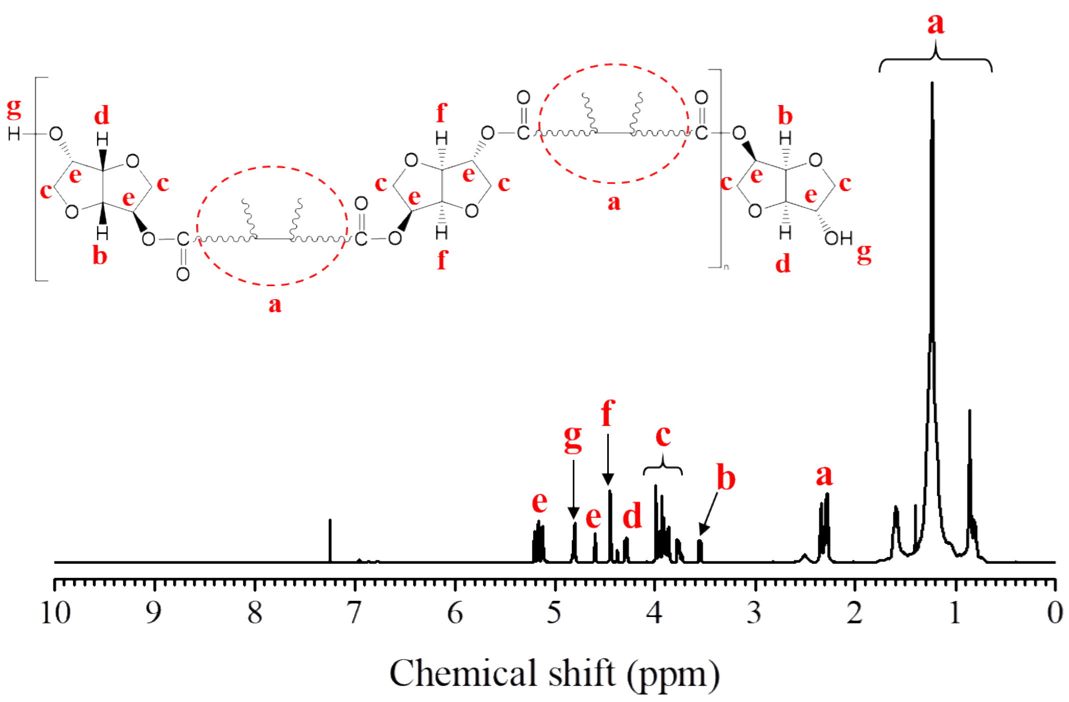

Figure S2. ^{1}H NMR spectrum of fully biobased polyester polyol based on ISB and DA (DIS).

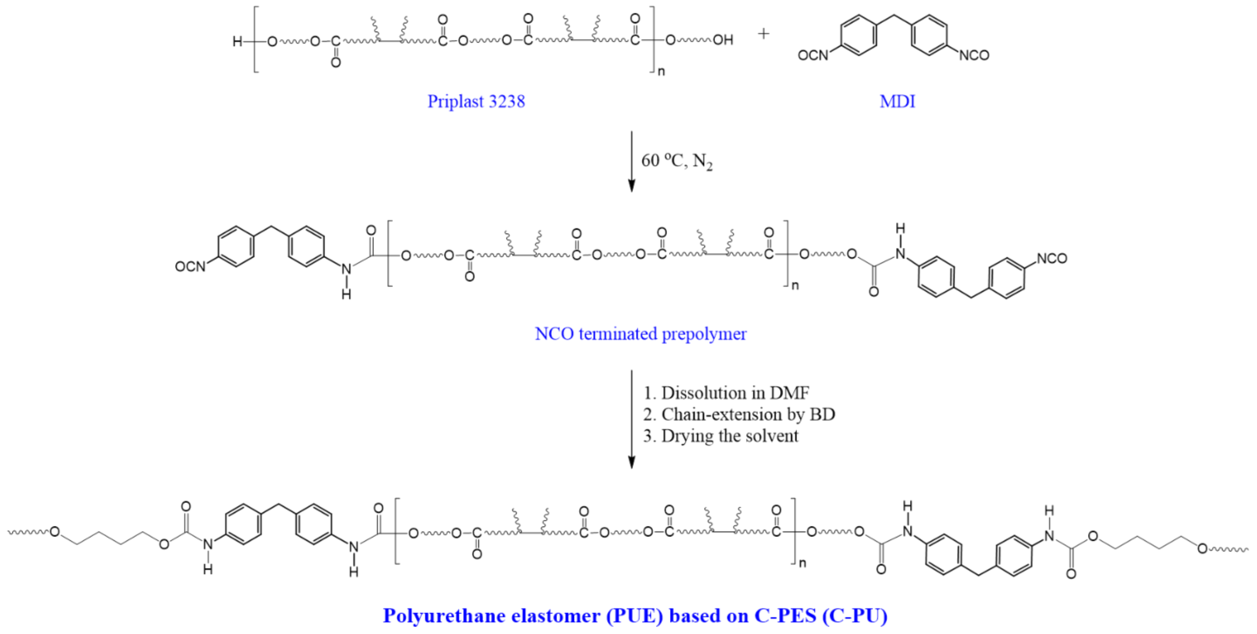

Figure S3. Synthetic route of PUE based on C-PES (Priplast 3238) (C-PU).

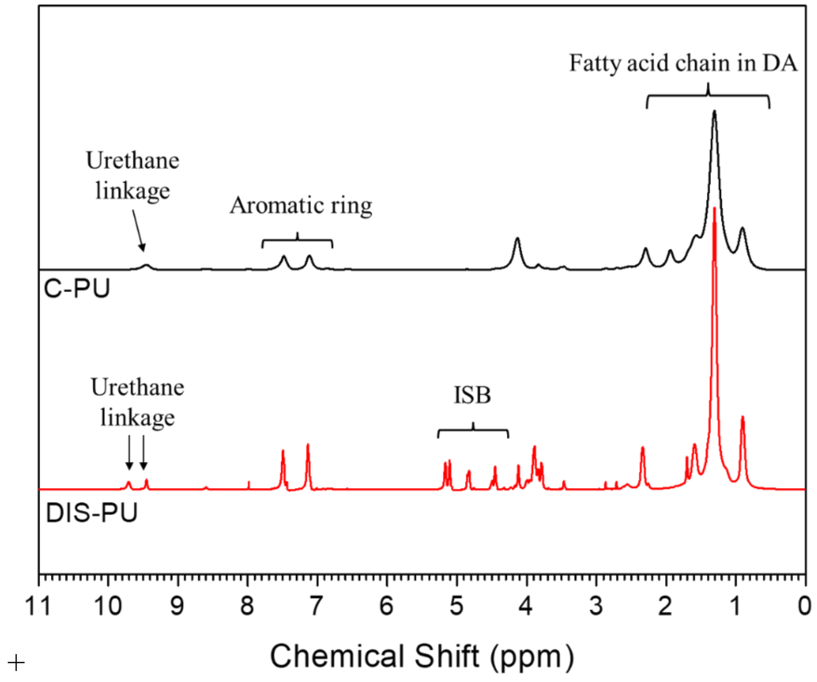

Figure S4. ^{1}H NMR spectra of DIS-PU and C-PU.

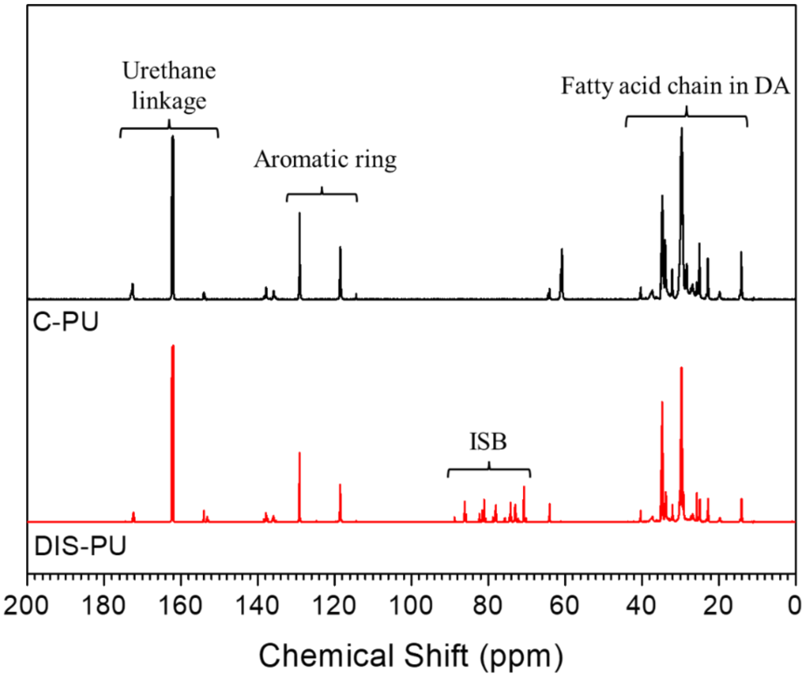

Figure S5. ^{13}C NMR spectra of DIS-PU and C-PU.

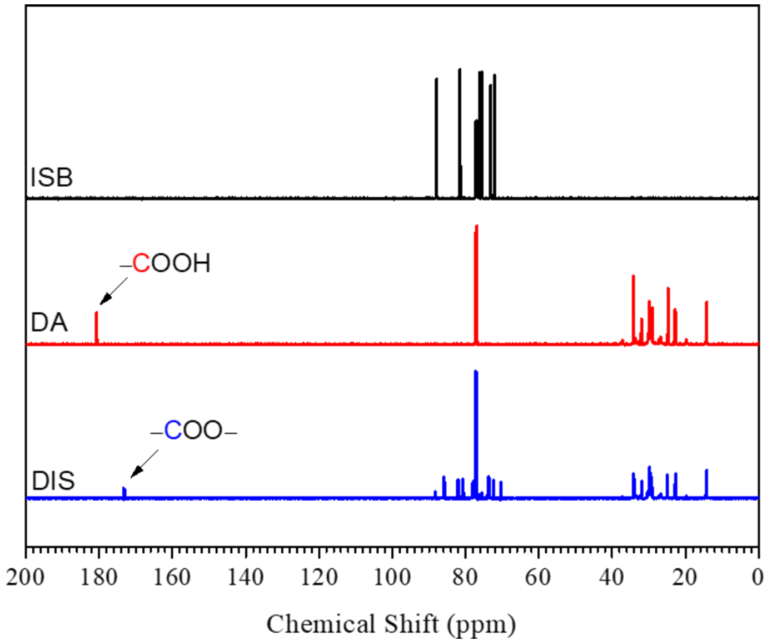

Figure S6. ^{13}C NMR of ISB, DA, and DIS.

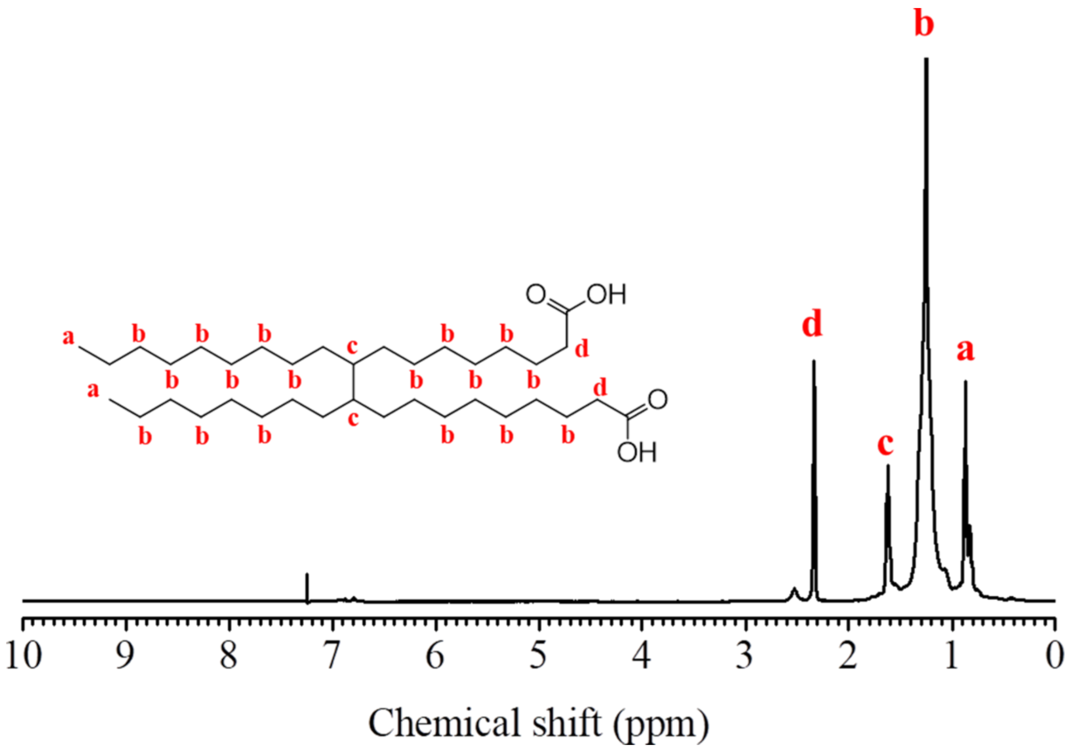

Figure S7. ^{1}H NMR spectrum of DA in $CDCl_3$.

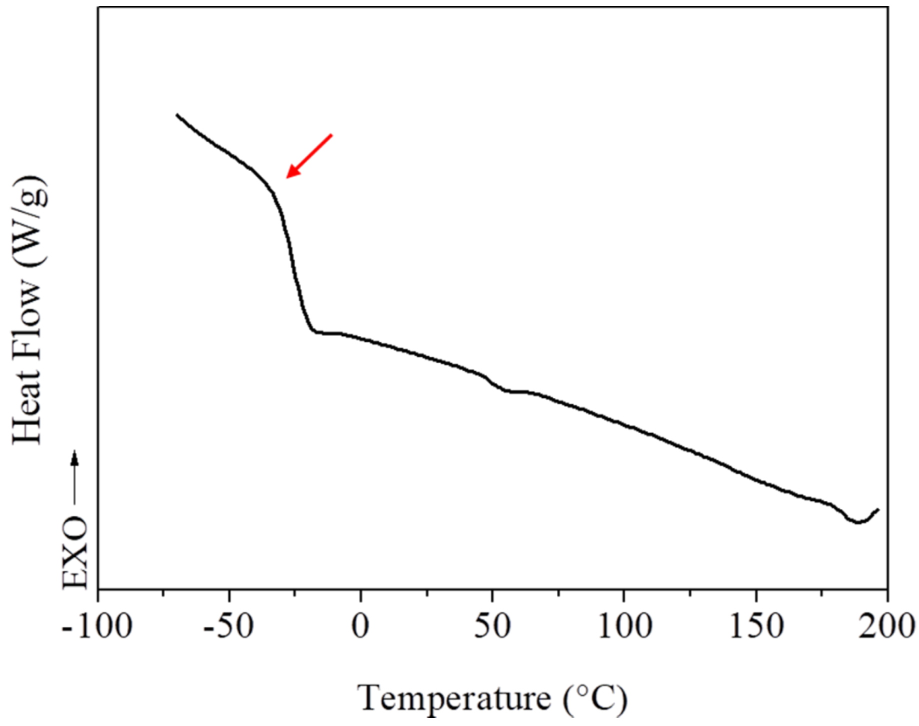

Figure S8. DSC thermogram of DIS under N_2 atmosphere.

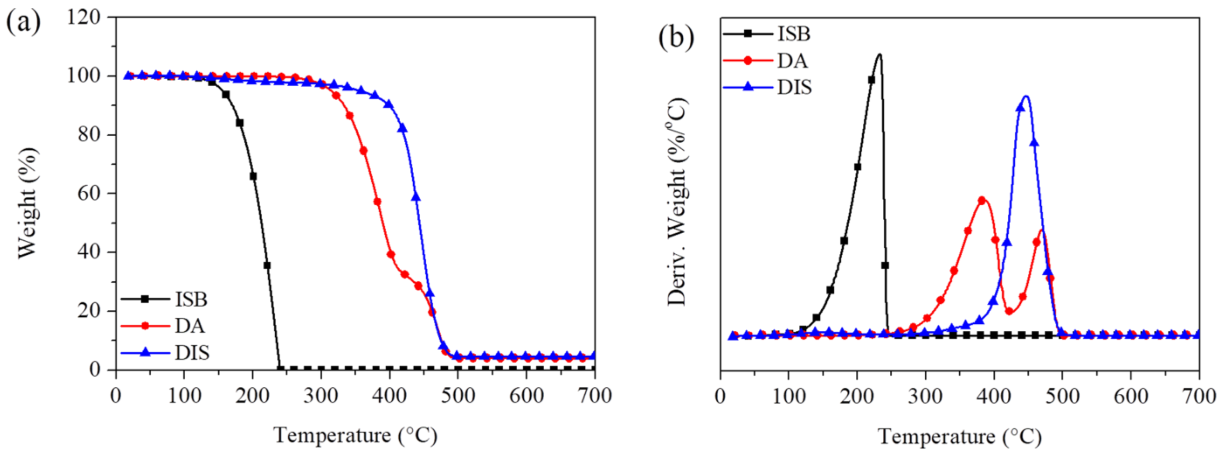

Figure S9. (a) TG and (d) DTG thermograms of ISB, DA, DIS at N_2 atmosphere.

Table S1. Characteristic temperatures of DIS-PU and C-PU determined by DSC and DMA

Sample	DSC			DMA
	$T_{g,ss}$	$T_{g,hs}$	$T_{m,hs}$	T_g
DIS-PU	-15.9	44.1	116.6	-16.2
C-PU	-44.7	47.9	119.3	18.3

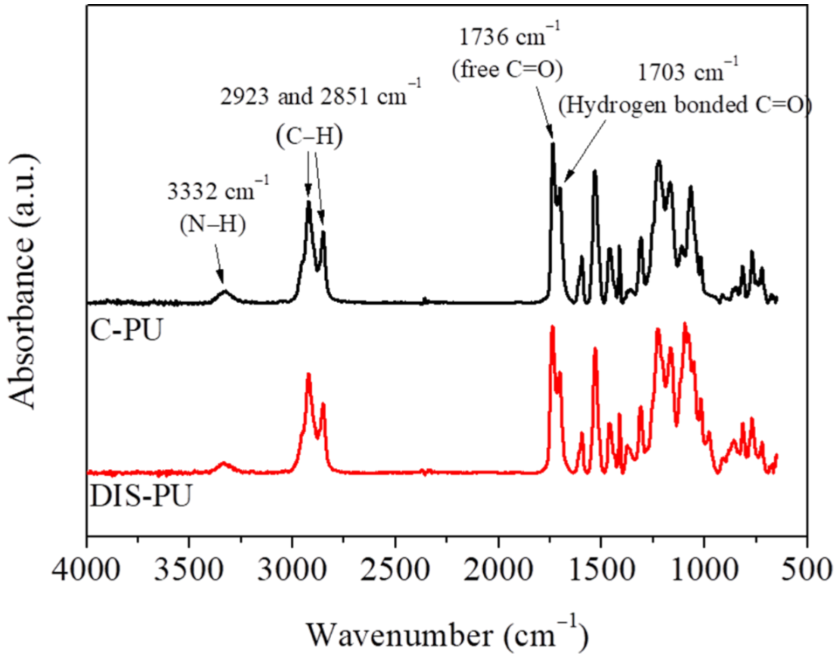

Figure S10. FTIR spectra of DIS-PU and C-PU measured employing ATR mode.

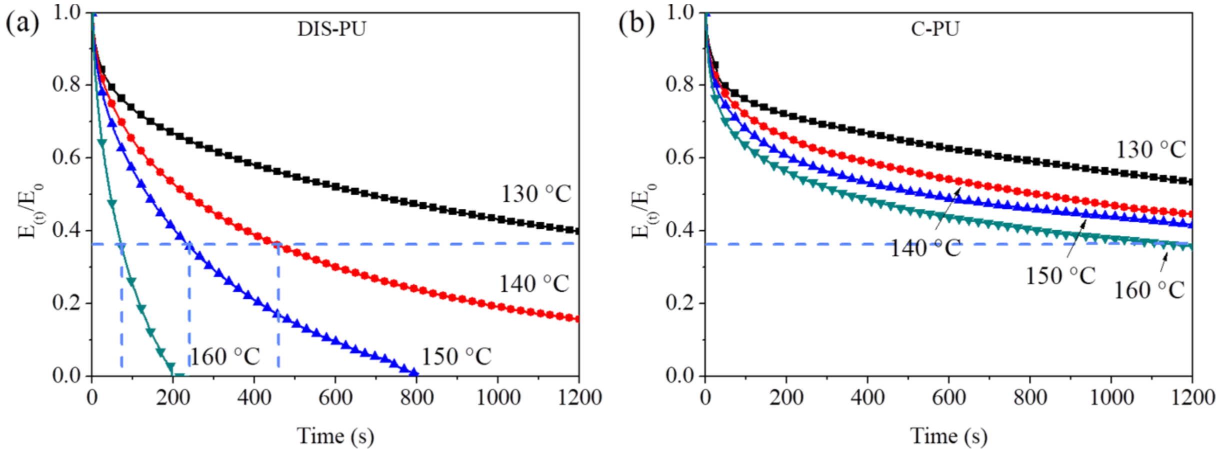

Figure S11. Normalized relaxation modulus ($E_{(t)}/E_0$) of (a) DIS-PU and (b) C-PU at different temperature.

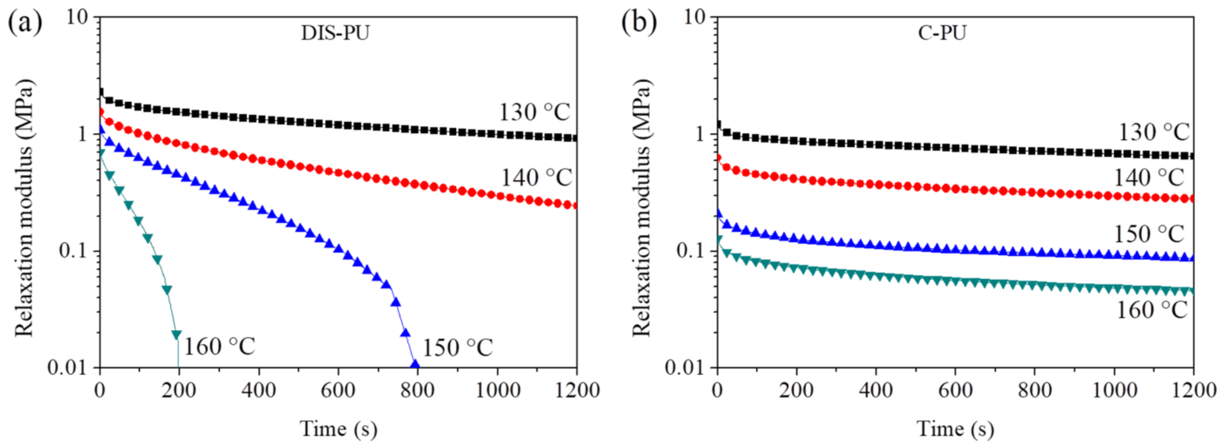

Figure S12. Stress relaxation modulus of DIS-PU and C-PU at different temperature.

Table S2. Stress relaxation time (τ(t)) of DIS-PU and C-PU at different temperature

Sample	τ(t) (s)			
	130	140	150	160
DIS-PU	1382	438	228	69
C-PU	-	-	-	1089

Table S3. Tensile strength and Elongation at break of DIS-PU and C-PU at different healing time

Sample		Healing Time (h)			
		Original	1 h	3 h	6 h
Tensile Strength (MPa)	DIS-PU	8.2±0.2	7.5±0.3	8.0±0.2	8.1±0.1
	C-PU	5.3±0.1	2.9±0.2	3.3±0.4	3.4±0.1
Elongation at break (%)	DIS-PU	173.2±8.0	150.4±9.6	194.2±5.8	236.8±11.1
	C-PU	654.6±7.5	227.8±14.3	301.1±9.8	343.6±10.6

Table S4. Tensile strength and Elongation at break of DIS-PU and C-PU at different repeated cycles of cutting and healing

Sample		Number of Repeated Test			
		Original	1st cycle	2nd cycle	3rd cycle
Tensile Strength (MPa)	DIS-PU	8.2±0.2	8.0±0.1	7.6±0.2	6.8±0.3
	C-PU	5.3±0.1	3.4±0.1	3.5±0.2	3.3±0.1
Elongation at break (%)	DIS-PU	173.2±8.0	236.8±11.1	137.5±14.6	87.4±13.2
	C-PU	654.6±7.5	343.6±10.6	341.1±11.2	309.6±10.1

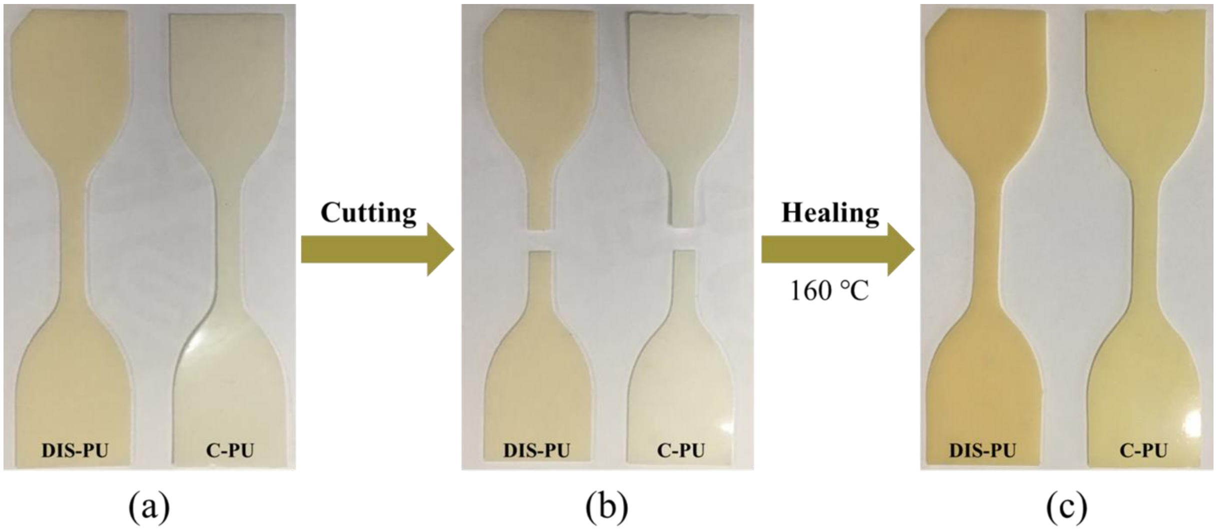

Figure S13. Photographs of dog-bone-shaped specimens of DIS-PU and C-PU and route for cutting & healing test to obtain self-healing efficiency.

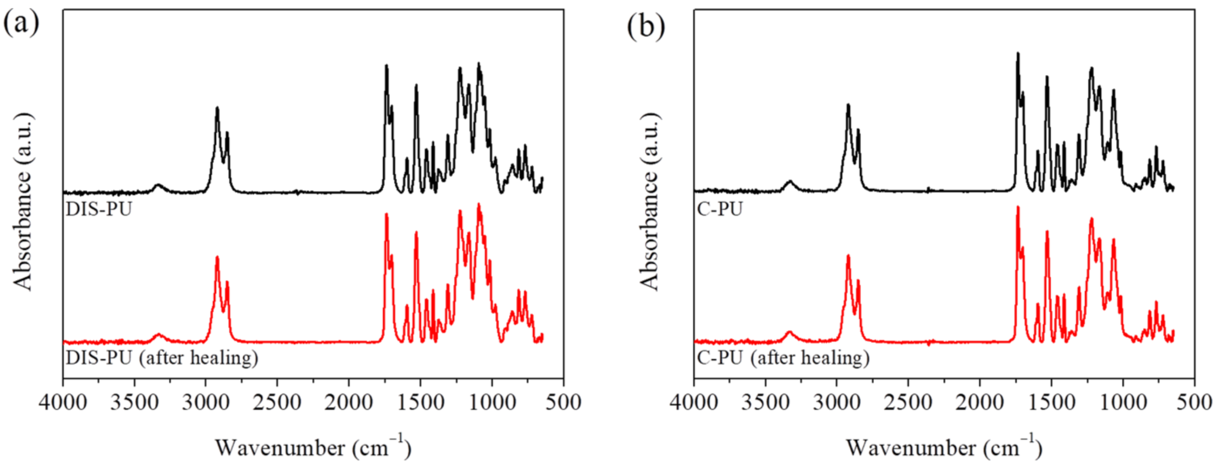

Figure S14. Comparisons of FTIR spectra of both DIS-PU and C-PU before and after self-healing at 160 °C for 6 h.

Table S5. The number of molecular weight (M_n) and polydispersity index (PDI) of DIS-PU and C-PU after self-healing at 160 °C

Sample	M_n (g/mol)	PDI
DIS-PU	13,130	3.26
C-PU	17,120	2.26

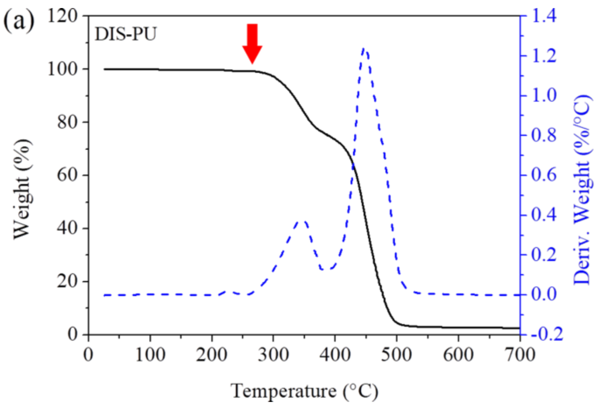

Figure S15. TG and DTG thermograms of (a) DIS-PU and (b) C-PU at N_2 atmosphere.

Table S6. Characteristic decomposition temperatures of DIS-PU and C-PU

Sample	$T_{5\%}$[a] (°C)	$T_{10\%}$[b] (°C)	T_{max1}[c] (°C)	T_{max2}[d] (°C)	Residue (%)
DIS-PU	314	332	345	449	2.5
C-PU	321	341	331	438/476	1.3

[a]$T_{5\%}$ indicates the decomposition temperature at 5 wt% of weight loss. [b]$T_{10\%}$ indicates the decomposition temperature at 10 wt% of weight loss. [c]T_{max1} indicates the maximum decomposition temperature of first decomposition stage. [d]T_{max2} indicates the maximum decomposition temperature of second decomposition stage.

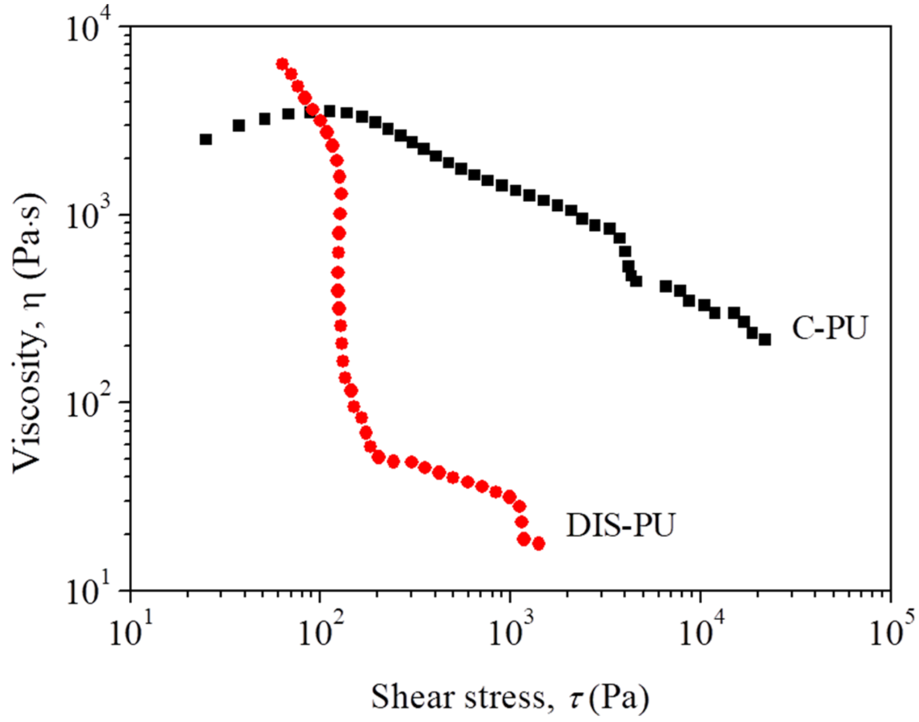

Figure S16. Shear viscosity versus shear rate of DIS-PU and C-PU at 180 °C.

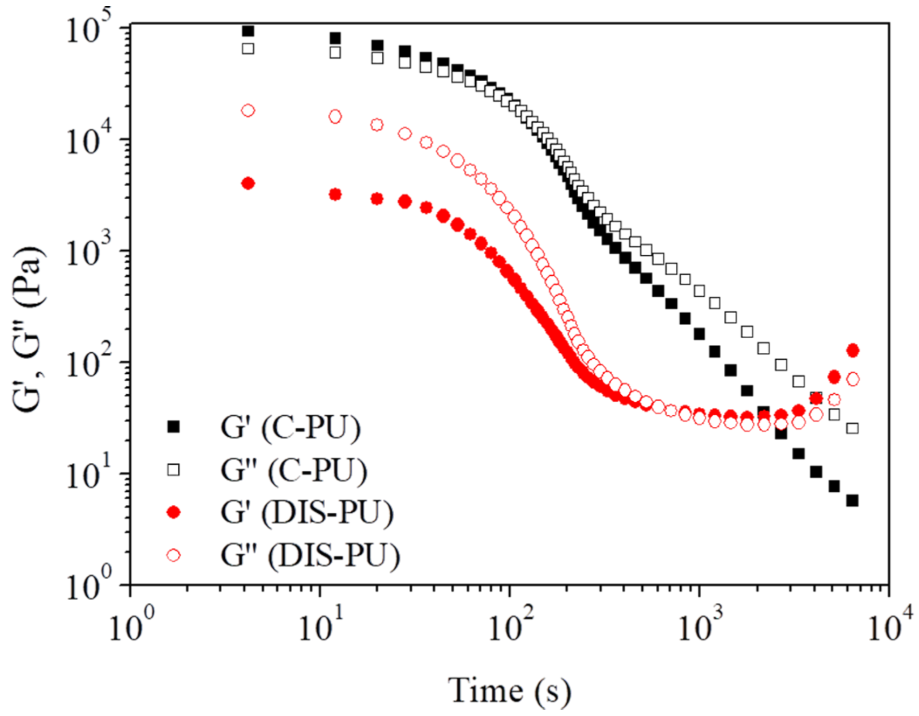

Figure S17. Storage modulus (G′) and loss modulus (G″) as a function of time of DIS-PU and C-PU at 180 °C.

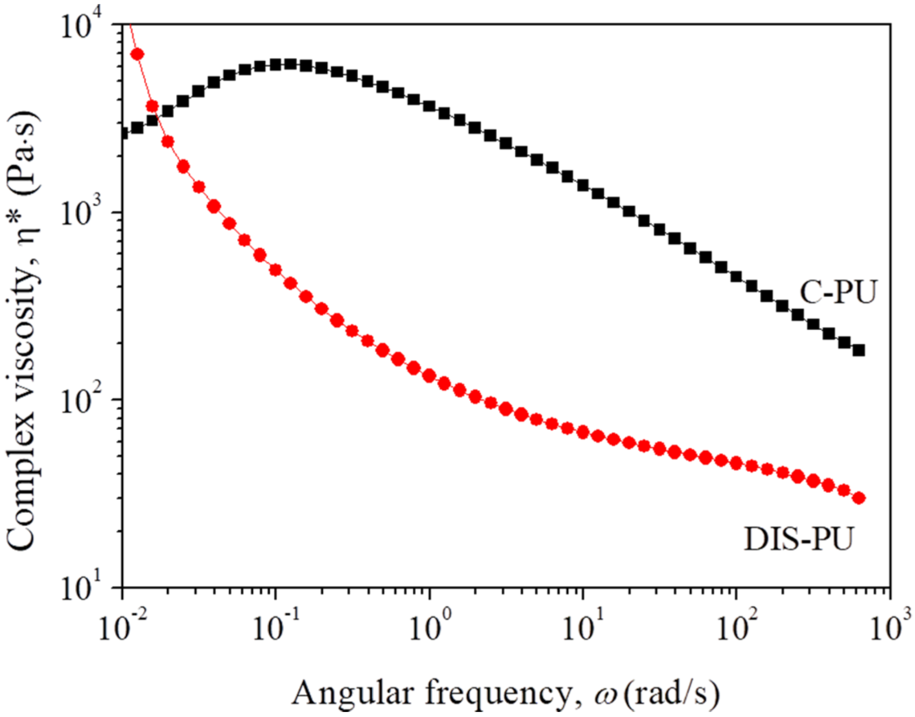

Figure S18. Complex viscosity (η*) versus angular frequency of DIS-PU and C-PU at 180 ℃.
